# Maternally inherited diabetes and A3243G mitocondrial mutation in a Brazilian kindred: diagnosis after clinical suspicion in an internal medicine unit

**DOI:** 10.1186/1758-5996-7-S1-A207

**Published:** 2015-11-11

**Authors:** Frederico Lisboa Nogueira, Livia Villela Costa, Nadine Márcia de Faria, Henry Houlden, Daniel Dutra Romualdo Silva

**Affiliations:** 1 Hospital Alberto Cavalcanti-Fhemig, Brazil

## Background

Maternally inherited diabetes and deafness (MIDD) is a rare cause of diabetes (DM) occurring due to mutation in mitochondrial DNA. The A3243G substitution in the tRNA leucine gene is the most common mutation and is found in 0.4% of those with type 2 DM. The suspicion and diagnosis are important given the unique management issues and associated comorbidities of this disease.

## Objective

Raise the awareness of monogenic diabetes, presenting the features that led to clinical suspicion in a patient with MIDD.

## Materials and methods

Case report.

## Results

We report a case of a 44-year's old woman admitted to the Internal Medicine ward with a two weeks history of signs and symptoms of community-acquired pneumonia and heart failure. She was known to have DM since the third decade of life, hearing loss since the adolescence in addition to hypertension and was in use of metformin, losartan, simvastatin and NPH insulin, with recurrent episodes of hypoglycemia. Physical examination showed a 23Kg/m^2^ BMI. Muscular weakness and ptosis were absent. Ophthalmological examination was unremarkable. She had a low C peptide (0.34ng/mL) and a glycated hemoglobin of 6.7%. Audiogram revealed severe bilateral neurossensorial loss mainly in high frequencies. Echocardiogram showed an enlarged atrium (42mm), a low ejection fraction (43%) with lateral and inferolateral hypertrophy. Familial history was noteworthy: his son died at 21 because of high undiagnosed hyperglycemia. Three of four brothers also had DM and one additionally had deafness and history of stroke and seizures, raising the suspicion of MELAS syndrome. Blood sample were collected from the kindred for molecular testing and using specific primers to the relevant region of mtDNA bp 3558-3539 and bp 3130-3149 PCR amplification was carried out. The A3243G mutation was identified in blood leukocytes with 70% heteroplasmy in the proband and with variable percentage in the family.

## Conclusion

The diagnosis of mitochondrial diabetes relies on high clinical suspicion to select those who benefits from molecular analysis. Systemic features like hearing loss, cardiomyopathy, myopathy and neurological symptoms associated with a strong familial history must call the attention for the diagnosis. This allows appropriate therapeutic management of hyperglycemia, early detection and treatment of associated disorders in addition to genetic counselling.

* Written informed consent was obtained from the patient for publication of this abstract.

